# Efficacy of true cinnamon (*Cinnamomum verum*) leaf essential oil as a therapeutic alternative for *Candida* biofilm infections

**DOI:** 10.22038/ijbms.2021.53981.12138

**Published:** 2021-06

**Authors:** Gayan Kanchana Wijesinghe, Thaís Rossini de Oliveira, Flávia Camila Maia, Simone Busato de Feiria, Janaina Priscila Barbosa, Felipe Joia, Giovana Cláudia Boni, José Francisco Höfling

**Affiliations:** 1 Area of Microbiology and Immunology, Department of Oral Diagnosis, Piracicaba Dental School, State University of Campinas, SP, Brazil

**Keywords:** Antifungal agent, Biofilms, Candida spp., Cinnamomum verum, Essential oil

## Abstract

**Objective(s):**

The essential oil (EO) extracted from *Cinnamomum verum* leaves has been used as an antimicrobial agent for centuries. But its antifungal and antibiofilm efficacy is still not clearly studied. The objective of this research was to evaluate the *in vitro* antifungal and antibiofilm efficacy of C. *verum* leaf EO against C. *albicans*, C. *tropicalis*, and C. *dubliniensis* and the toxicity of EO using an *in vitro* model.

**Materials and Methods:**

The effect of EO vapor was evaluated using a microatmosphere technique. CLSI microdilution assay was employed in determining the Minimum Inhibitory (MIC) and Fungicidal Concentrations (MFC). Killing time was determined using a standard protocol. The effect of EO on established biofilms was quantified and visualized using XTT and Scanning Electron Microscopy (SEM), respectively. Post-exposure intracellular changes were visualized using Transmission Electron Microscopy (TEM). The toxicological assessment was carried out with the Human Keratinocyte cell line. The chemical composition of EO was evaluated using Gas Chromatography-Mass Spectrometry (GC-MS).

**Results:**

All test strains were susceptible to cinnamon oil vapor. EO exhibited MIC value 1.0 mg/ml and MFC value 2.0 mg/ml against test strains. The killing time of cinnamon oil was 6 hr. Minimum Biofilm Inhibitory Concentration (MBIC_50_) for established biofilms was <0.2 mg/ml for all test strains. SEM images exhibited cell wall damages, cellular shrinkages, and decreased hyphal formation of *Candida*. TEM indicated intracellular vacuolation, granulation, and cell wall damages. Cinnamon leaf oil caused no inhibition of HaCaT cells at any concentration tested. Eugenol was the abundant compound in cinnamon oil.

**Conclusion:**

C. *verum* EO is a potential alternative anti-*Candida* agent with minimal toxicity on the human host.

## Introduction

The use of medicinal plants as a treatment option is an ancient practice and continues in the modern world. Ancient Asians including Sri Lankans, Indians, and Japanese people especially used these phytochemicals as their therapeutic agents. The use of medicinal plants as therapeutic alternatives has helped many populations that do not have any access to novel treatments and drugs of high cost and low availability. So, the use of plants for the treatment of diseases/infections has been empirically employed worldwide. In the past few years, with scientific advances, more research was conducted to introduce new compounds with medicinal properties, allowing scientists to find new, effective, alternative medicinal compounds with low side effects (1-4).

Essential oils extracted from *C. verum* leaves and bark were used as antimicrobial agents for centuries. Though this plant has been shown to be of great economic and pharmaceutical-medicinal interest (5), proper evidence-based studies are not yet carried out to evaluate the efficacy of cinnamon oils as an antimicrobial therapeutic agent.

Biofilms are communities of microorganisms attached to a surface living in a matrix of extracellular material derived both from the cells themselves and from the environment. Those microorganisms are usually organized into a three-dimensional structure (6-8) in order to form this complex biofilm ecosystem. Many human microbial infections (more than 65%) are related to biofilm formation on implanted biomaterials and/or host surfaces (9, 10). Biofilms are more resistant to most available antimicrobials compared with planktonic counterpart. Thus, it is important to introduce alternative biofilm controlling and eradication strategies in order to treat biofilm infections (11).

*Candida *spp. causes many oral, non-oral, and device-associated biofilm infections including periodontitis, dental caries (12), and endocarditis (13), systemic candidiasis, superficial *Candida* skin infections, ear infections, genitourinary tract infections, central venous catheters, prosthetic heart valves, and urinary catheter associated infections (14-16). Some of these infections are serious life-threatening infections while some are non-invasive and non-life-threatening.

Although several *in vitro *microbiological studies with essential oil extracted from *C. verum* leaves show antimicrobial activities related to many bacterial species in the health care area, including those that exhibit multiple antimicrobial resistance, its antifungal and antibiofilm effects on *Candida* spp. are still not comprehensive.

## Materials and Methods

***Fungal strains***

Three *Candida* type strains, *C. albicans* (ATCC MYA-2876), *C. tropicalis* (ATCC 750), and *C. dubliniensis* (ATCC MYA-646) were used in this study. These strains were obtained from Microbiology and Immunology Area, Piracicaba Dental School, UNICAMP, Brazil. 

The standard *Candida* stocks were maintained in 80% glycerol in an ultrafreezer at -80 ^°^C. To reactivate stock organisms, they were subcultured in freshly prepared Saboraud Dextrose Agar (SDA, OXOID) culture medium and incubated aerobically at 37 ^°^C for 24 hr.

The standard cell suspensions were prepared by adjusting the turbidity in accordance with the 0.5 McFarland scale, which was equivalent to the absorbance of 0.08–0.10 (600 nm) corresponding to 5×10^6^ CFU/ml.

All experiments were done in triplicate on two different occasions. 

***Essential oil***

The essential oil of *C. verum* leaves extracted using steam distillation was purchased from Romik Lanka Marketing Services, Moratuwa, Sri Lanka (WCC/3569). The *C. verum* essential oil was diluted to 32 mg/ml in Tween 80 (0.05%) solution and Roswell Park Memorial Institute (RPMI) 1640 buffered with MOPS (3-(N-morpholino) propane sulfonic acid) followed by sonication, 1 cycle of 20 sec for antimicrobial assays and *in vitro* cytotoxicity. (17). 

***Effect of C. verum leaf essential oil in the vapor phase***

Effects of *C. verum* leaf essential oil in vapor phase on planktonic *Candida* cells were qualitatively determined using the microatmosphere method described by Şerban *et al*. (2011) (18).

Standard suspensions of all test strains were prepared as explained previously and SDA plates were inoculated separately using a sterile cotton swab. A sterile filter paper disc moistened with the working solution (100 µl) of EO was attached to the lid of the Petri dish. Plates were sealed with a piece of parafilm and incubated overnight at 37 ^°^C aerobically. Control plates were prepared without filter paper disks. The presence or absence of growth inhibition of inoculated *Candida* on agar surface was observed after 24 hr incubation.

***Determination of minimum inhibitory concentration (MIC) and minimum fungicidal concentration (MFC)***

According to Clinical and Laboratory Standards Institute (CLSI) M27-A3 (19), 100 µl/well of the two-fold dilutions of EO in RPMI 1640 with MOPS were prepared in 96-well polystyrene microtiter plates. Subsequently, 100 µl of the prepared standard fungal cell suspensions containing 1×10^6^ cells/ml were added to the dilutions and incubated at 37 ^°^C for 24 hr. The MIC point was determined by visual observation for the presence and absence of growth (turbidity of the suspensions).

MFC was defined as the lowest concentration of essential oil required to inhibit the fungal growth completely. For MFC determination, 5 μl of solutions from each well of the previous experiment was plated in freshly prepared SDA plates and incubated at 37 ^°^C for 24 hr aerobically. The lowest concentration which did not show any *Candida* growth on the SDA surface after incubation was considered as MFC.

Negative control group (Growth control): 100 µl of RPMI 1640 instead of essential oil+standard cell suspension. Positive control group: 120 mg/ml chlorhexidine digluconate (Sigma-Aldrich, USA). 

***Killing time assay***

One hundered µl of *C. albicans* (ATCC MYA-2876) (0.5×10^6^ CFU/ml) was mixed with 100 µl of 1.0, 2.0, and 4.0 mg/ml of EO respectively to obtain the final EO concentrations of MIC (1 mg/ml), half of MIC (0.5 mg/ml), and twice the MIC (2 mg/ml or MFC). After different incubation time intervals (0, 1, 2, 3, 4, 5, 6, 12, and 24 hr) at 37 ^°^C, 50 µl of each mixture was diluted in sterile normal saline, plated on SDA using a sterile glass spreader, and incubated at 37 ^°^C for 24 hr. Colonies were counted and CFU/ml was plotted against time (20) after 24 hr incubation.

Negative control: test organisms without oil or reference antifungal agent. 120 mg/ml chlorhexidine digluconate: positive control.

***Minimum biofilm inhibitory concentration (MBIC***_50_ ***)***

A 96-well sterile flat-bottomed polystyrene microplate was seeded with 5×10^6^ CFU/ml standard inoculum of each test organism (100 μl/well) followed by aerobic incubation for 24 hr at 37 ^°^C. The plate was washed once with sterile Phosphate Buffered Saline (PBS), and 100 μl of the essential oil dilutions were added to the treatment wells separately. 100 μl of RPMI 1640 was added to the negative control wells instead of oil. The plate was then aerobically incubated for 24 hr at 37 ^°^C. Biofilm viability was quantified using XTT assay (17, 21).

***Minimum biofilm eradication concentration (MBEC)***

The CFU assay was performed on EO-treated biofilms to detect the MBEC of the oil on established 24 hr mature biofilms after the 24 hr treatment with different concentrations of *C. verum* leaf oil, as explained previously (17). 

***Post-exposure architecture of established Candida biofilms (scanning electron microscopy (SEM)***

For determination of post-exposure architectural properties of *Candida* biofilms, mature biofilms were established on sterile 10 mm diameter glass coverslips and treated with 0.5, 1.0, and 2.0 mg/ml EO dilutions in RPMI 1640 for 24 hr as explained in a previous experiment and processed to examine under SEM (1, 22). 

***Transmission electron microscopy (TEM) for determination of post-exposure cellular changes***

To determine the effect of *C. verum* leaf oil on *Candida *cell structure, post-exposure cellular changes were visualized using a Transmission Electron Microscope (TEM) as follows.

Briefly, standard cell suspensions with 1×10^6^ cell density were prepared in RPMI 1640. 1 ml of prepared suspensions were mixed with 9 ml of antifungal oil dilution in RPMI 1640 with the concentration of 10×MIC (10 mg/ml) separately (final EO concentration of the mixture was 1.0 mg/ml) and incubated at 37 ^°^C for 24 hr aerobically. After incubation, the resulting cell suspension was centrifuged and the cell pellet was resuspended with the Karnovsky’s fixative for 18-24 hours at 4 ^°^C. After fixation, the specimen was processed as explained previously by Kapoor *et al*. (2017) (23).

***Cytotoxicity of C. verum leaf oil on human cells***

Cytotoxicity of *C. verum* leaf EO on normal human keratinocyte cell line, HaCaT was determined by MTT (3-(4,5-dimethylthiazol-2-yl)-2,5-diphenyl-2H-tetrazolium bromide) viability assay according to the protocol described by Zanette *et al.* (2011) (24). 

Briefly, 96-well culture plates were seeded with cells (6.5×10^4^ cells/ml and 100 µl/well) and incubated for 24 hr. Then the culture medium was replaced with fresh medium containing different concentrations of true cinnamon leaf EO. The treated plates were further incubated for 24 hr at 37 ^°^C with 5% CO_2_. After the incubation, the viability of treated HaCat cells was quantified using an MTT assay (25). 

***Chemical analysis of the essential oil***

The chemical analysis of *C. verum* essential oil was performed by using the following gas chromatography-mass spectrometry (CG-EM) analysis conditions. 

HP-6890 gas chromatograph coupled with HP-5975 selective mass detector; HP-5MS Capillary Column (30 m×0.25 mm×0.25 µm); temperatures: injector (220 ^°^C), detector (250 ^°^C), column (60 ^°^C ), 3 ^°^C/min, 240 ^°^C ; flow rate of carrier gas (highly dried He) of 1.0 ml/min.

***Statistical analysis***

Statistical analysis was carried out using the software, Statistical Package for Social Sciences (SPSS) version 16. Multiple means of more than three data sets were compared using one-way ANOVA and two-way ANOVA. The level of significance was taken at 5% (*P*<0.05).

## Results

***Anti-Candida effect of C. verum leaf oil vapor***

The results for antifungal efficacy of true cinnamon leaf oil vapor against three *Candida* test strains are presented in Table 1. Presence of growth inhibition on the agar surface indicates the sensitivity of relevant microorganisms to the treatment (18). All *Candida *test strains exhibited growth inhibition on the agar surface which is indicator of antifungal activity of *C. verum* leaf EO vapor on planktonic *Candida* (Table 1).

***Minimum inhibitory concentration (MIC) and minimum fungicidal concentration (MFC)***

The MIC and MFC values corresponding to *C. verum* EO and the control antifungal chlorhexidine digluconate are represented in Table 2. *C. verum* exhibited similar efficacy as chlorhexidine digluconate on planktonic *C. albicans* (ATCC MYA-2876) and *C. dubliniensis* (ATCC MYA-646). *C tropicalis* (ATCC 750) was more susceptible for chlorhexidine digluconate compared with *C. verum* leaf oil. The emulsifier (Tween 80) did not affect the growth of the fungal strains at the used concentration. The MFC/MIC ratio showed that the *C. verum* leaf EO had a fungicidal effect on all three* Candida* species tested.

***Killing time assay***

**Figure 1:**
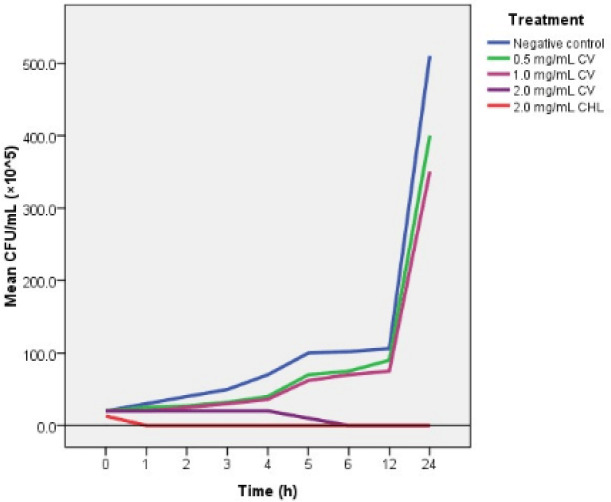
Killing curves of *Candida* *albicans* (ATCC MYA-2876) for 0.5 mg/ml, 1.0 mg/ml, and 2 mg/ml *C.* *verum* leaf oil and 2.0 mg/ml chlorhexidine digluconate. Error bars are too small to indicate

This experiment determined the minimum time required for true cinnamon leaf oil (or positive control chlorhexidine digluconate) to eradicate the viable planktonic *Candida* cells from their *in vitro *cultures. Killing curves for *C. albicans* (ATCC MYA-2876) were presented in Figure 1. 

According to obtained results, 0.5 mg/ml and 1.0 mg/ml *C. verum* leaf oil do not kill *Candida* cells completely within 24 hr test period, whereas 2.0 mg/ml kill *Candida* cells completely within 6 hr. 

2.0 mg/ml chlorhexidine digluconate has a rapid killing action on *Candida* cells compared to true cinnamon leaf oil hence killing time was 1 hr. 

***Minimum biofilm inhibitory concentration (MBIC***_50_ ***)***

Minimum concentrations of the essential oil required to reduce the biofilm cell viability by 50% of negative control biofilms (biofilms without treatments) were defined as MBIC_50_. 

**Figure 2:**
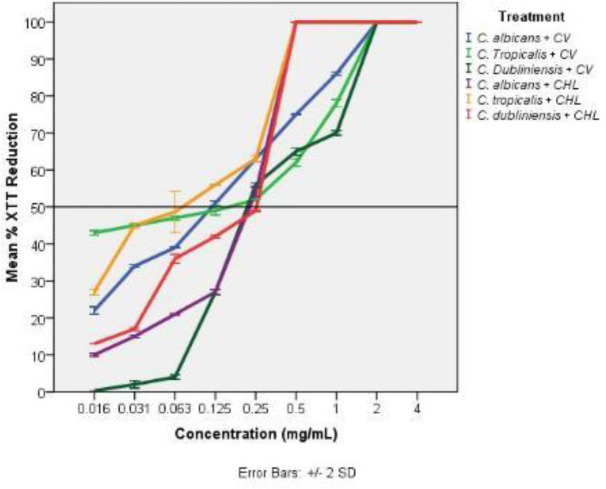
Percentage reduction of XTT metabolic activity of *Candida albicans* (ATCC MYA-2876), *C.* *tropicalis* (ATCC 750), and *C. dubliniensis* (ATCC MYA-646) 24 hr established biofilms after treatment with different concentrations of *Cinnamomum verum* leaf oil (CV) and chlorhexidine digluconate (CHL). All error bars represent ± 2 standard deviations (SD)

Figure 2 shows the percentage reduction of biofilm cell viability of 24 hr mature *C. albicans* (ATCC MYA-2876), *C. tropicalis* (ATCC 750), and *C. dubliniensis* (ATCC MYA-646) biofilms determined by XTT viability assay after 24 hr treatment with *C. verum* leaf oil compared with the negative control (biofilms without treatment).

According to obtained data from the XTT assay, concentrations required to reduce the biofilm viability by 50% are shown in Table 3.

***Minimum biofilm eradication concentration (MBEC)***

**Figure 3:**
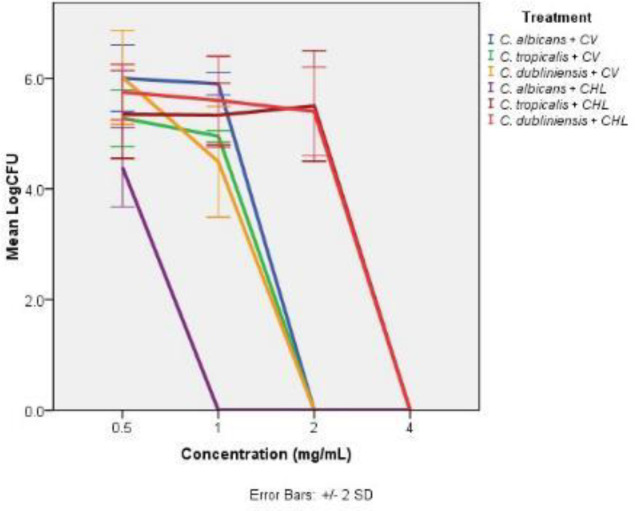
Mean Log CFU values of established *Candida albicans* (ATCC MYA-2876), *C. tropicalis* (ATCC 750), and *C. dubliniensis* (ATCC MYA-646) biofilms after 24 hr treatment with different concentrations of CV: *Cinnamomum verum* leaf oil and CHL: chlorhexidine digluconate All error bars represent the ± 2 standard deviations (SD)

The minimum concentration of *C.verum* leaf oil required to kill the 24 hr mature biofilm completely (MBEC) was determined using CFU assay. Figure 3 represents the viability of 24 hr mature biofilms after treating with different concentrations of *C. verum* leaf oil and chlorhexidine digluconate for 24 hr determined by CFU assay. Table 4 shows MBEC values for mature *C. albicans* (ATCC MYA-2876), *C. tropicalis* (ATCC 750), and *C. dubliniensis* (ATCC MYA-646) biofilms.

***Scanning electron microscopy (SEM) of established biofilms***

**Figure 4:**
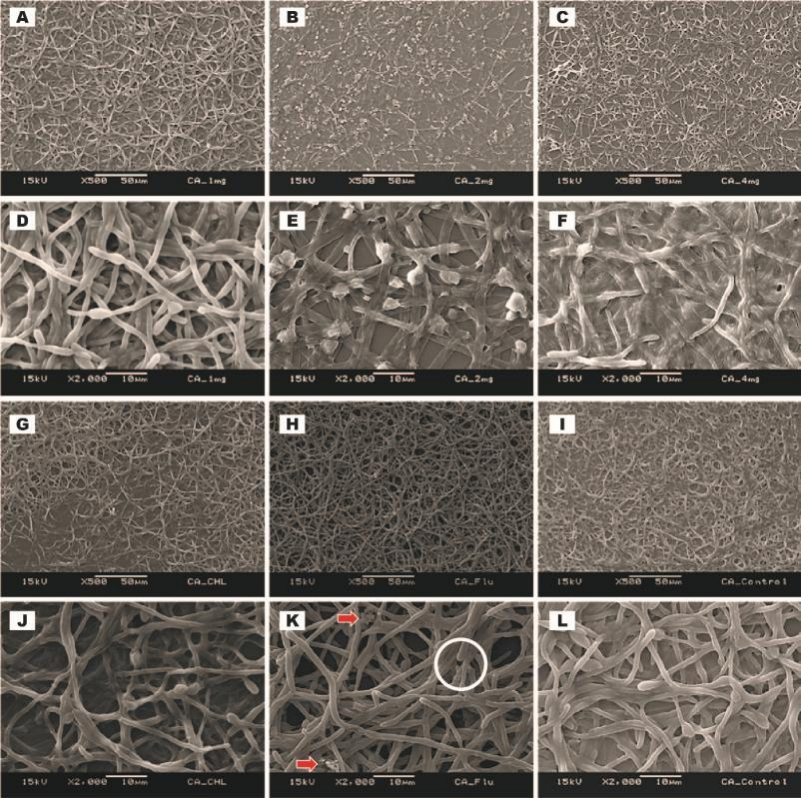
SEM images of *Candida albicans* (ATCC MYA-2876) established biofilms after 24 hr treating with 1.0 mg/ml *Cinnamomum verum* leaf oil (A and D), 2.0 mg/ml *C. verum* leaf oil (B and E), 4 mg/ml *C. verum* leaf oil (C and F), 1.0 mg/ml chlorhexidine digluconate (G and J) and 0.008 mg/mL Fluconazole (H and K). I and L: Negative control. Circles- Cell wall deformities with treatments. Red solid arrows- Leakages of intracellular components

**Figure 5:**
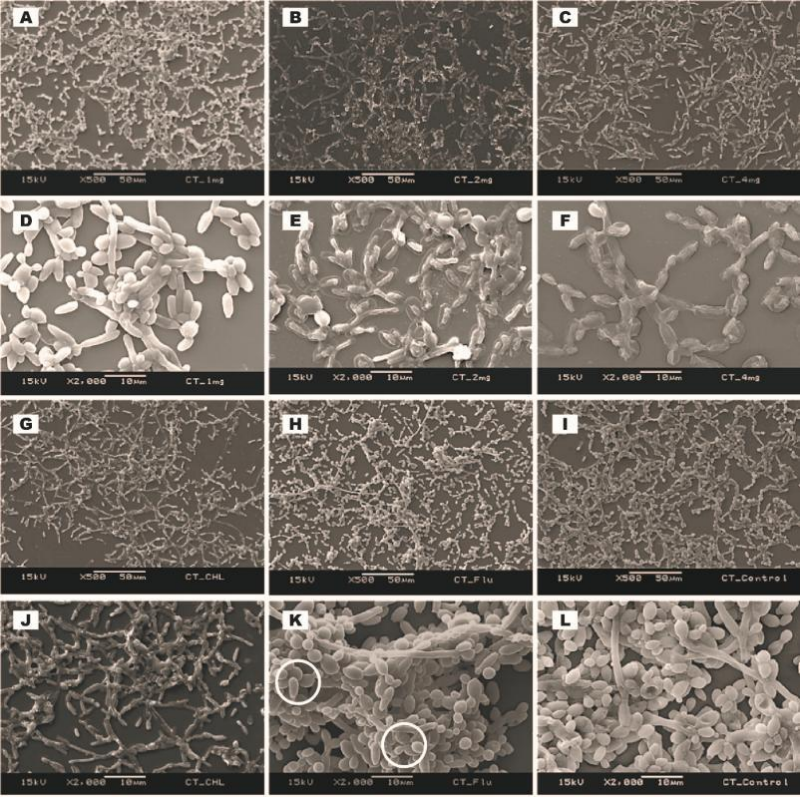
SEM images of *Candida tropicalis* (ATCC 750) established biofilms after 24 hr treatment with 1.0 mg/ml *Cinnamomum verum* leaf oil (A and D), 2.0 mg/ml *C. verum* leaf oil (B and E), 4.0 mg/ml *C. verum* leaf oil (C and F), 4.0 mg/ml chlorhexidine digluconate (G and J) and 0.008 mg/ml Fluconazole (H and K) I and L: Negative control. Solid circles indicate Cell wall deformities with treatments

**Figure 6:**
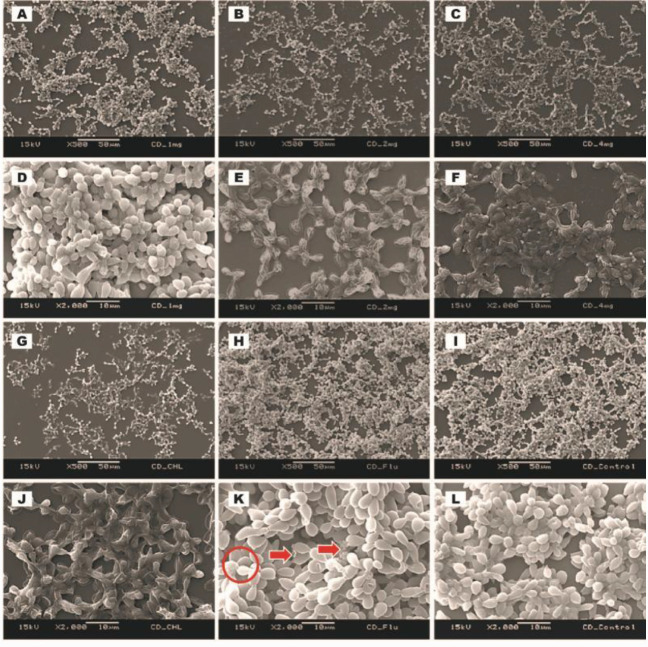
SEM images of *Candida dubliniensis* (ATCC MYA-646) established biofilms after 24 hr treatment of 1.0 mg/ml *Cinnamomum verum* leaf oil (A and D), 2.0 mg/ml *C. verum* leaf oil (B and E), 4.0 mg/ml *C. verum* leaf oil (C and F), 4.0 mg/ml chlorhexidine digluconate (G and J), and 0.008 mg/ml Fluconazole (H and K) I and L: Negative control. Solid circles-Cell wall deformities with treatments. Solid arrows-Leakages of intracellular components

Ultrastructure of established biofilms of *C. albicans* (ATCC MYA-2876), *C. tropicalis* (ATCC 750), and *C. dubliniensis* (ATCC MYA-646) after treating with 1.0, 2.0, and 4.0 mg/ml of *C. verum* leaf oil, 1 mg/ml (for *C. albicans* biofilms), 4.0 mg/ml (for *C. tropicalis* and *C. dubliniensis* biofilms) chlorhexidine digluconate and 0.008 mg/ml Fluconazole was qualitatively evaluated by SEM (Figures 4, 5, and 6).

*C. verum* leaf oil caused *Candida* cell shrinkage by damaging walls of mature sessile cells, pseudohyphae, and hyphae and caused leakage of intracellular materials. These effects were concentration-dependent. Maximum cell damage was observed with 4 mg/ml of *C. verum* leaf oil. Both 2 mg/ml and 4 mg/ml of *C. verum* leaf oil showed complete destruction of 24 hr mature biofilms of *C. albicans* (ATCC MYA-2876), *C. tropicalis* (ATCC 750), and *C. dubliniensis* (ATCC MYA-646) with 24 hr treatment. 

When considering chlorhexidine digluconate, it exhibited a similar effect as *C. verum* leaf oil (damaging cell walls of *Candida *cells and causing cytoplasmic leakages) on established *Candida* biofilms. Similar observations were obtained for 24 hr established biofilms of *C. albicans* (ATCC MYA-2876), *C. tropicalis* (ATCC 750), and *C. dubliniensis* (ATCC MYA-646) with 0.008 mg/ml Fluconazole. Biofilms treated with 0.008 mg/ml Fluconazole exhibited rough cellular surfaces indicating that cell wall deformities and leakages of intracellular components were observed in *C. albicans *and *C. dubliniensis* biofilms.

***Transmission*** ***electron microscopy (TEM)***

Post-exposure cellular morphology of planktonic *C. albicans* (ATCC MYA-2876); *C. dubliniensis* (ATCC MYA-646), and *C. tropicalis* (ATCC 750) was determined using TEM. 

**Figure 7:**
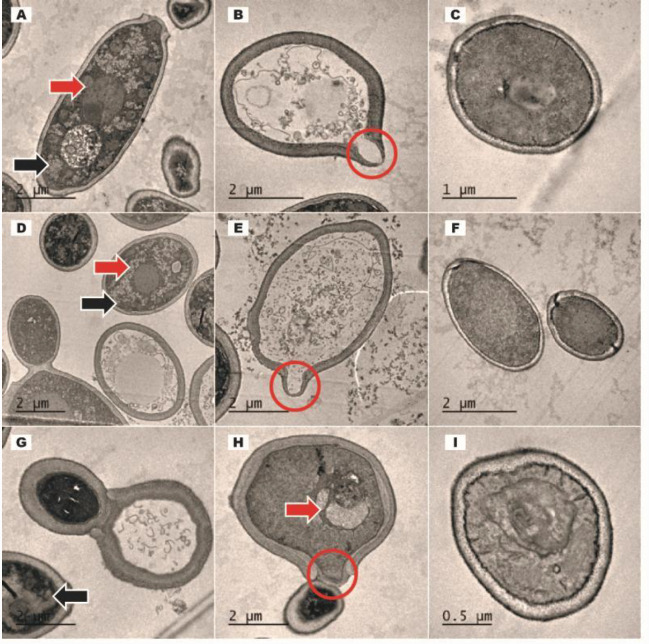
Transmission Electron Microscopic (TEM) images of *Candida albicans* (A, B, and C); *C. tropicalis* (D, E, and F) and *C. dubliniensis* (G, H, and I). Red solid arrows indicate intra-cellular vacuoles, red circles indicate cell wall damages, and black solid arrows indicate cytoplasmic coarse granular inclusion bodies. C, F, and I are negative controls

TEM images of test strains after 24 hr exposure to MIC (1.0 mg/ml) of *C. verum* leaf oil and chlorhexidine digluconate were obtained as follows (Figure 7): TEM clearly indicates cell wall damages, intracellular granulations, and vacuole formation.

***In vitro*** **cytotoxicity of** ***C. verum*** **leaf oil (HaCaT human keratinocyte cell line)**

**Figure 8:**
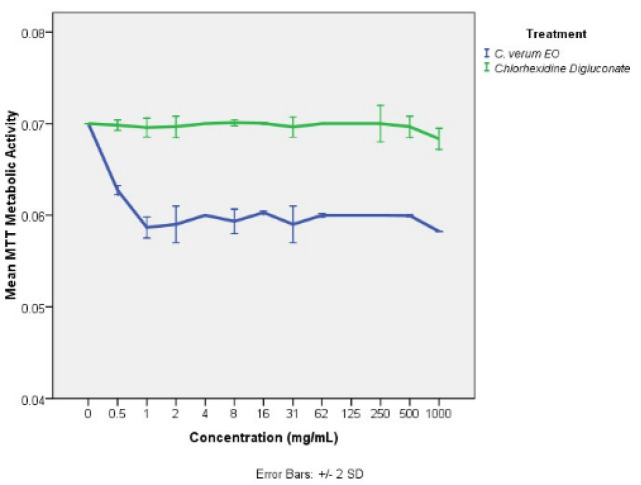
Post-exposure MTT metabolic activity/viability of HaCaT cell line after 24 hr treatment with different concentrations of *Cinnamomum verum* leaf oil and chlorhexidine digluconate. All error bars represent the ± 2 standard deviations (SD)

Figure 8 shows percentage of growth inhibition after treatment with different concentrations. No significant change in HaCaT cell viability within the 0-1000 mg/ml concentration range of true cinnamon leaf EO or chlorhexidine digluconate was detected. In this experiment, no toxic effect on HaCaT cells was noted with any concentrations of EO tested.

***Chemical composition of true cinnamon leaf oil***

The most abundant compound of *C. verum* leaf EO was Eugenol (77.22%). Benzyl benzoate (4.53%), trans caryophyllene (3.39%), acetyle eugenol (2.75%), and linalool (2.11%) were identified as minor components.

**Table 1:** Inhibition zones exhibited by planktonic test strains in the presence of working concentration (1.0 g/ml) of *Cinnamomum verum* leaf oil. All experiments were done in triplicate

**Organism**		Presence (+) /Absence (-) of growth inhibition	Sensitive/Resistant
	*C. albicans* (ATCC 5314)	+	Sensitive
	*C. tropicalis* (ATCC 750)	+	Sensitive
	*C. dubliniensis* (ATCC MYA-646)	+	Sensitive

**Table 2:** Results of MIC and MFC of *Candida* spp. The experiment was made in triplicates with three individual experiments. Chlorhexidine digluconate was used as a positive control

Organism		*Cinnamomum verum *oil (mg/mL)			Chlorhexidine digluconate (mg/mL)				
		MIC	MFC	MFC/MIC	MIC	MFC		MFC/MIC	
	*C. albicans* (ATCC 5314)	1.0	2.0	2.0	1.0		2.0		2.0
	*C. tropicalis* (ATCC 750)	1.0	2.0	2.0	0.5		0.5		1.0
	*C. dubliniensis* (ATCC MYA-646)	1.0	2.0	2.0	1.0		2.0		2.0

CV: C. *verum* leaf oil, CHL: Chlorhexidine digluconate

**Table 3:** Minimum biofilm inhibitory concentrations (MBIC_50_) for established biofilms of *Candida albicans* (ATCC MYA-2876), C. *tropicalis* (ATCC 750), and C. *dubliniensis* (ATCC MYA-646)

	*C. albicans*		*C. tropicalis*		*C. dubliniensis*	
	*C. verum *	CHL	*C. verum *	CHL	*C. verum *	CHL
MBIC_50_ (mg/mL)	0.1	0.2	0.2	0.0625	0.2	0.3

CHL: Chlorhexidine digluconate

**Table 4:** Concentrations of *Cinnamomum verum* leaf oil and chlorhexidine digluconate that kill the established biofilms of *Candida albicans* (ATCC MYA-2876), *C. tropicalis* (ATCC 750), and *C. dubliniensis* (ATCC MYA646) completely (MBEC)

Test strain	MBEC (mg/mL)	
	*C. verum* leaf oil	Chlorhexidine digluconate
*C. albicans* (ATCC MYA-2876)	2.0	1.0
*C. tropicalis* (ATCC 750)	2.0	4.0
*C. dubliniensis* (ATCC MYA-646)	2.0	4.0

## Discussion

Cinnamon has been used as a common culinary spice in several communities for centuries. Additionally, cinnamon has been also employed as folk medicine and phytomedicinal alternative in different cultures. The present study was conducted to find out the efficacy of *C. verum*/ true cinnamon leaf oil as a potential phytomedicinal therapeutic alternative against three commonest infection-causing *Candida *strains, namely *C. albicans* (ATCC MYA-2876), *C. tropicalis* (ATCC 750), and *C. dubliniensis* (ATCC MYA-646). The results of the present study show that *C. verum *EO had an important antifungal activity on all the tested strains of *Candida*.

In the present study, observations obtained from the microatmospheric plate method showed anti-*Candida* activity of *C. verum* leaf oil vapor. All three test strains exhibited a zone of growth inhibition with the administration of *C. verum* oil vapor (Table 1). Since, at normal body temperature, *C. verum *leaf oil vaporizes, it is important to evaluate the efficacy of an antimicrobial agent when it is not directly in contact with the affected areas. So this step can be considered as a screening test for anti-*Candida *activity of *C. verum* EO in its vapor phase. 

The MFC/MIC ratio suggests that true cinnamon leaf EO had a fungicidal effect (26) on all three test strains. Though these data, in principle, demonstrate the antifungal action of *C. verum* leaf oil on planktonic *Candida,* MIC and MFC values of the current study are slightly different from recently published findings. (27, 28). These differences in MIC and MFC may be due to the concentration variations of active compounds in EO used. 

According to the classification of the activity level of plant materials based on MIC value introduced by Duarte *et al.* (2005), MIC up to 0.5 mg/ml is considered as strong active, concentrations from 0.55 to 1.5 mg/ml are considered as moderately active, and above 1.5 mg/ml are considered as weakly active plant materials against tested microorganisms (29). According to this classification, the results obtained with the EO of *C. verum* leaves showed moderate antifungal activity on planktonic *Candida*. On the other hand commercial antifungal chlorhexidine digluconate exhibited a similar anti-*Candida *action as *C. verum* leaf oil for *C. albicans* and *C. dubliniensis* by demonstrating MIC value of 1 mg/ml (Table 2). 

As well as minimum effective concentrations, the time required to completely eradicate/kill the microbial population is one of the key determinants of antimicrobial potency of a given antimicrobial agent and plays a major role in designing the dosage regime of a given antimicrobial drug (30). Results obtained from the killing time assay of *C. verum* leaf oil on test strain exhibited a concentration-dependent killing time. MFC (2.0 mg/ml) of cinnamon kills *C. albicans* within 6 hr whereas 2.0 mg/ml chlorhexidine digluconate kills *C. albicans* within 1 hr (Figure 1). Sub-MFC concentrations of *C. verum* EO does not exhibit any fungicidal effect within the 24 hr experiment period. Since killing time is concentration-dependent, shorter killing times can be acquired by increasing the concentration of EO, but the toxicological assessments should be contemplated. Though killing time is considered as an essential component of the antimicrobial profile, there is no evidence-based data on time-kill kinetics of *C. verum* leaf EO on *Candida* spp. The present study fills this deficiency of available data with an *in vitro * experimental design performed on ATCC type *Candida* strains. 

In addition to the ability of true cinnamon leaf EO to act on *Candida* spp. planktonic cells, a strong antifungal potential was detected relative to the 24 hr established *Candida* biofilms. True cinnamon EO at a concentration of 0.2 mg/ml reduced the viability of *C. tropicalis* and *C. dubliniensis* biofilms by at least 50%, except for the *C. albicans* biofilm, which exhibited a 50% viability reduction with 0.1 mg/ml EO concentration (Table 3). These findings suggest the potential use of *C. verum* leaf EO as an antifungal for patients affected with chronic candidiasis. Notably, 24 hr exposure of 2 mg/ml EO caused complete killing of mature biofilms of the three types of investigated biofilms (Table 4). Though there are few studies in the literature on the antimicrobial effect of *C. verum *leaf EO, no published study assessed its action on *Candida spp.* biofilms. 

SEM images were taken to understand the structure of biofilms after subjecting to EO chemical stress. All biofilms of test strains exhibited cell wall damages, cell wall deformities, and leakages of intracellular materials with treatment of *C. verum* leaf oil (Figures 4, 5, and 6). Importantly, scanning electron microscopy confirmed the dose-dependent nature of the effects of cinnamon leaf EO. Fluconazole is a known antifungal agent which belongs to the azole group and inhibits synthesis of fungal sterol, ergosterol (20). On the other hand, chlorhexidine is a biguanide that is used as an antibacterial mouth rinse. It alters the morphology of cells, damages the cell wall of microorganisms, and releases intracellular components. It has been suggested as a well-known therapeutic antifungal agent for oral candidiasis (31, 32). Since both antimicrobial agents have an effect on *Candida* cell wall, the ultrastructure of established *Candida* biofilms with MBEC of chlorhexidine digluconate and 0.008 mg/ml Fluconazole (maximum recommended *in vitro* assay concentration, CLSI) was also visualized. The intensity of the post-exposure response of Fluconazole was minimal due to low concentration. 

To confirm the SEM observations, and to evaluate the intracellular/morphological changes of unicellular yeast forms, TEM images of *C. albicans* (ATCC MYA-2876), *C. tropicalis* (ATCC 750), and *C. dubliniensis* (ATCC MYA-646) test strains were taken after treatment with MIC of *C. verum* oil. TEM images showed cell wall damages and leakages of the intracellular compartment (Figure 7). Importantly, TEM images revealed post-exposure cytoplasmic changes including intracellular vacuoles formation, scattered cytoplasm, and formation of cytoplasmic granular inclusion bodies. These changes are suggestive of chemical stress on *Candida* cells by *C. verum* leaf oil.

The toxicity of true cinnamon EO on host tissues was evaluated using an *in vitro *cell culture model of the human non-cancer keratinocytes (HaCaT) cell line. Toxicology assessments provide proper understanding of effective, nontoxic dose and help in dose regimen designing by integrating both pharmacodynamics and pharmacokinetics of *C. verum* leaf oil. Furthermore, there is a lack of published data on toxicology studies of *C. verum* leaf oil and the current study fills that deficiency. The maximum concentration tested in this study was 1000 mg/ml (neat concentration of true cinnamon leaf EO oil) and none of the tested concentrations showed any inhibitory effect on HaCaT cells (Figure 8). This finding indicates the safe use of *C. verum* leaf EO up to 1000 mg/ml without any toxic effect on human cells. 

Based on *in vitro *toxicology study results and pharmacodynamics of *C. verum* leaf oil on *Candida* sessile and planktonic cells, true cinnamon leaf EO can be considered a potential therapeutic alternative for *Candida *infections. Also, the authors suggest future research on the effect of *C. verum* leaf EO on a wide range of pathogenic fungi and bacteria.

The results of the chemical analysis of the true cinnamon leaf EO agree with the findings by previous authors, which indicate that eugenol is its most abundant chemical component (77.22%) (33, 34) whereas the other chemical compounds appear in smaller concentrations.

According to available data, a similar mode of antifungal action of both cinnamon leaf oil and chlorhexidine digluconate was noted. Lipophilic eugenol can interfere with cell wall integrity and fluidity as well as biofilm extracellular matrix by actively penetrating the phospholipid bilayer of the cell membrane and ultimately disrupting it (35). 

## Conclusion

The EO extracted from *C. verum* leaves contains eugenol as its major chemical component. It possess an antifungal action on *Candida* spp. planktonic cells and biofilm by acting on the cell wall integrity. Moreover, it does not have any toxic effect on human keratinocytes.
